# Recent Advances in the Structure, Extraction, and Biological Activity of *Sargassum fusiforme* Polysaccharides

**DOI:** 10.3390/md23030098

**Published:** 2025-02-23

**Authors:** Shun Zhang, Liang Chen, Nan Shang, Kefeng Wu, Wang Liao

**Affiliations:** 1 Key Laboratory of Environmental Medicine and Engineering of Ministry of Education, and Department of Nutrition and Food Hygiene, School of Public Health, Southeast University, Nanjing 210009, China; 220234056@seu.edu.cn; 2 Public Service Platform of South China Sea for R&D Marine Biomedicine Resources, The Marine Biomedical Research Institute, Guangdong Medical University, Zhanjiang 524023, China; cliang@gdmu.edu.cn (L.C.); winvee@gdmu.edu.cn (K.W.); 3 College of Engineering, China Agricultural University, Beijing 100083, China; nshang@cau.edu.cn; 4 Key Laboratory of Precision Nutrition and Food Quality, Department of Nutrition and Health, China Agricultural University, Beijing 100083, China

**Keywords:** *Sargassum fusiforme* polysaccharides, structure, extraction methods, biological activity

## Abstract

*Sargassum fusiforme* polysaccharides (SFPs) are acidic polysaccharides that possess significant medicinal and commercial potential. This review aims to summarize recent advances in the structure, extraction methods, and diverse biological activities of SFPs, including their antioxidant, antitumor, immunomodulatory, antiviral, intestinal flora-regulating, and anti-diabetic properties. The key findings reveal the complex composition of polysaccharides, highlighting alginic acid, fucoidan, and laminaran as the primary constituents, and detailing their structural features. At the same time, the characteristics as well as the advantages and disadvantages of hot water extraction, acid extraction, alkali extraction, ultrasonic extraction, microwave extraction, and enzyme extraction were systematically compared. Finally, this review concludes by emphasizing the necessity for further research to elucidate the structure–function relationships of SFPs, optimize their extraction techniques, and provide a theoretical foundation for subsequent studies.

## 1. Introduction

*Sargassum fusiforme* (SF), also known as Hizikia fusiforme, belongs to the family Sargassaceae in the Phaeophyta [1]. SF is predominantly found in the northwestern Pacific Ocean, where it grows abundantly along the coastal regions of China, Japan, and Korea [2]. SF is abundant in nutritional benefits and can be used for both food and medicinal purposes. As early as in ancient China, the medicinal value of SF has been documented in Shennong’s Herbal Classic and Compendium of Materia Medica [3]. Modern medical research has also demonstrated that SF possesses antioxidant, anti-tumor, and anti-inflammatory properties, as well as the ability to regulate blood sugar and enhance human immunity. Sargassum is a seaweed that is high in protein and low in fat, and it is rich in polysaccharides, dietary fiber, vitamins, minerals, amino acids, and a variety of trace elements. As a medicinal and dietary algal plant, SF is also an excellent raw material for industrial processing, with broad medicinal and commercial value.

Polysaccharides account for 20~70% of the dry weight of SF, which is one of the important bioactive substances of SF [4]. SFPs have been found to possess antioxidant properties [5] and exhibit potential in the prevention and treatment of cancer [6]. Additionally, they help regulate the immune system and serve as hypoglycemic and hypolipidemic agents, aiding in the management of diabetes mellitus and its complications [7]. SFPs primarily consist of alginic acid and fucoidan, which constitute cell walls, along with a small amount of laminaran, found within the cytoplasm [8]. Alginic acid is a linear polymer formed by linking α-L-guluronic acid (G) and β-D-mannuronic acid (M) with 1→4 glycosidic bonds, which has the effect of promoting endothelial cell growth and reducing lipid uptake [9]. The G/M ratio in alginic acid may fluctuate depending on factors such as the source of the seaweed, the season, the growth stage, and the extraction process. Laminaran is a small neutral polysaccharide with a simple structure, mainly composed of a large number of β-D-glucose residues [10]. The main chain is usually a β-(1→3) glycosidic linkage with some β-(1→6) glycosidic linkages. Fucoidan, a more active component of the polysaccharide fraction, is a polysaccharide rich in fucose and sulfate groups found in brown algae and some marine invertebrates [11]. It usually connects L-fucose via α-(1→3) and α-(1→4) glycosidic linkages. Numerous studies have been conducted on fucoidan of different sources, which has diverse biological activities: anticoagulant and antithrombotic, antitumor, antiviral, and anti-inflammatory [12,13,14]. The biological activity of fucoidan is mainly due to its high degree of sulfation, in addition to a relationship with the unique polysaccharide structure and relative molecular weight.

The structure of polysaccharides contains primary, secondary, tertiary, and quaternary structures, and polysaccharides are the most complex biomolecules compared to the structures of macromolecules such as proteins and nucleic acids [15]. The primary structure of polysaccharides is fundamental, and studies mainly focus on the molecular weight, monosaccharide composition and ratios (including neutral and acidic sugars), the type and quantitative content of uronic acids, as well as the sugar residue conformations. The common extraction processes of *Sargassum fusiforme* polysaccharides include hot water extraction, acid extraction, alkali extraction, enzyme extraction, ultrasonic-assisted extraction, and microwave-assisted extraction. The choice of different extraction methods had different effects on each of these indicators. This review encompasses the literature on the chemical composition, extraction and purification processes, and biological activities of SFPs in recent years, in order to help researchers gain a deeper understanding of SFPs.

## 2. Methodology

We conducted a structured search across multiple databases, including PubMed, Web of Science, Scopus, and Google Scholar. The search was performed using the following keywords: Sargassum fusiforme polysaccharides, structure, extraction methods, biological activity, polysaccharides composition, etc. Original research articles and review papers published between the last five years were selected. A total of 3680 articles were initially retrieved. After removing duplicates and screening titles and abstracts, the screened references were further scrutinized. Additionally, relevant references were manually identified from the citations of these selected articles. Finally, 104 articles were included as references.

## 3. The Composition of SFPs

The composition of SFPs is exceedingly complex, and the polysaccharides may still contain numerous impurities even after the extraction and purification processes. SFPs are vital biologically active components found in SF, primarily consisting of alginic acid, fucoidan, laminaran, and a minor fraction of dietary fiber [16]. SFPs, a category of acidic polysaccharides, have been subjected to chemical composition analysis which has uncovered the presence of sulfated groups alongside a suite of other components, including total sugars, proteins, and uronic acids [17]. The levels of these components were typically determined using the barium chloride, phenol–sulfuric acid colorimetric, Bradford, and carbazole methods, respectively, with comparisons made to standards such as K_2_SO_4_, glucose, bovine serum albumin, and D-glucuronic acid [18]. Additionally, other methodologies exist for quantifying the chemical constituents of polysaccharides. For instance, some researchers employ the hydroxy biphenyl method for the measurement of uronic acids [19]. The hydroxy biphenyl method is suitable for determining glucuronic acid in plants, microorganisms, and natural polysaccharides, and is particularly effective for analyzing samples with a high glucuronic acid content, such as pectin.

The chemical composition of polysaccharides from SF varies depending on the measurement method and extraction purification processes chosen by the researcher. Of course, the environment, source, and harvesting season of SF may also fluctuate the measurement results. Seasonal changes can affect the growth and metabolic processes of seaweed, leading to fluctuations in the polysaccharide composition [20].

**Figure 1:**
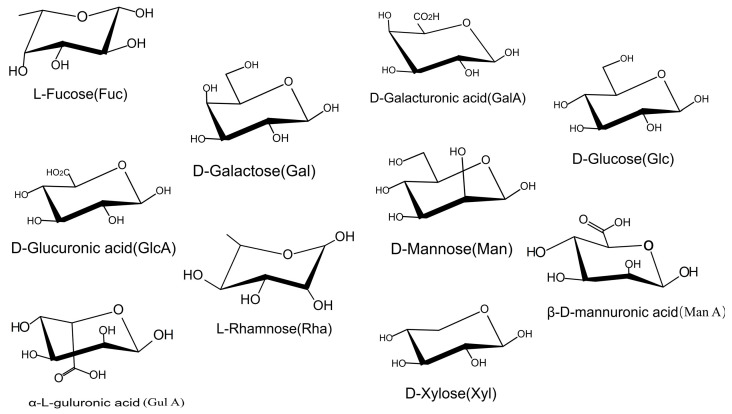
Structure of the monosaccharide fraction of the polysaccharides from SF. These monosaccharides can be classified based on their polysaccharide sources. Alginic acid: ManA, GulA. Fucoidan: Fuc, Gal, GalA, Rha, GlcA, Xyl. Laminaran: Glc, Man.

Molecular weight is usually analyzed using high-performance gel permeation chromatograghy (HPGPC), which is performed on a Waters 1000 HPLC system equipped with a Waters 2414 refractive index detector [21]. The initial calibration of the column was performed using dextran standards of different molecular weights. Analyzing the monosaccharide composition after hydrolysis of these polysaccharides using gas chromatography–mass spectrometry (GC/MS) [22] and high-performance liquid chromatography (HPLC) [14], and a novel method of high performance liquid chromatography/electrospray ionization mass spectrometry (HPLC-ESI-MS) [23] has revealed that they are primarily composed of fucose (Fuc), galactose (Gal), mannose (Man), glucose (Glc), xylose (Xyl), rhamnose (Rha), glucuronic acid (GlcA), galacturonic acid (GalA), mannuronic acid (ManA), guluronic acid (GulA), etc. The structure of each monosaccharide is shown in Figure 1. In addition to the natural factors, the choice of extraction and purification processes also affects the ratio of the aforementioned monosaccharides. The chemical compositions of the crude polysaccharides of SF are shown in Table 1. By quantitatively measuring the content of each component, Table 1 provides researchers with a scientific basis for future experimental design. Additionally, by analyzing the composition and molecular weight of the monosaccharides, the structure of a particular extraction product can be analyzed in depth in relation to its potential biological functions.

### 3.1. Fucoidan

**Figure 2:**
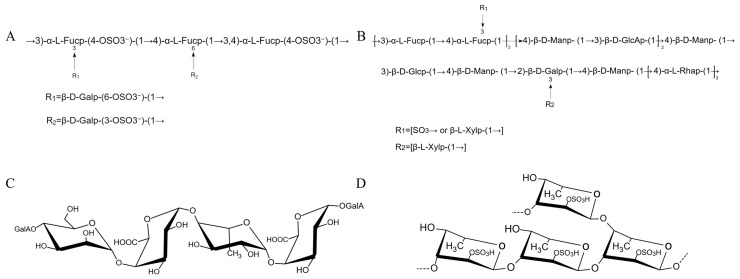
The predictive structure of fucoidan. (**A**) SFP, (**B**) SFF-32, (**C**) SFPS65-B, (**D**) F2-2.

Fucoidan, also known as fucoidan sulfate, is a naturally occurring water-soluble sulfated heteropolysaccharide found in brown algal interstitial tissues or mucus matrices, and is characterized by its richness in fucose and sulfate substituents [37]. The composition of fucoidan is relatively complex, the core structure is mainly sulfated fucoidan, accompanied by a small amount of galactose, xylose, rhamnose and other neutral sugars, glucuronic acid and galacturonic acid, and a small amount of protein and metal ions and other impurities. The total sugar content, relative molecular mass, sulfate group content, and structure of fucoidan may be affected by different extraction methods, different growth stages of SF and geographical locations. A more convincing experimental verification of the effects of different extraction methods on the polysaccharide fractions of fucoidan was provided by previous studies [13]. Typical fucoidan structures are shown in Figure 2.

According to the reported physicochemical properties of fucoidan (FP08S2), the percentage of chemical components in FP08S2 were sugar (16.8%), sulfate (20.8%), and glyoxalate (34.6%), respectively [37]. Its molecular weight was 47.5 kDa. After analyzing the composition of monosaccharides by gas chromatography, it was found that fucoidan consists of fucose (36.6%), mannose (7.0%), xylose (18.3%), galactose (19.1%), and glucuronic acid (19.1%). Furthermore, the absolute configurations of the constituent monosaccharides were identified as L-fucose, D-mannose, D-xylose, D-galactose, and D-glucuronic acid, respectively. This aligns with previous findings regarding the chiral molecules of fucoidan [38]. Fucoidan derived from SF possesses a glucuronomannan backbone, primarily composed of alternating →2)-α-D-Manp-(1→ and →4)-β-D-GlcAp-(1→ residues. The external branches are predominantly →3)-α-L-Fucp-(1→ residues, which are highly sulfated on C-2 and C-4, or terminal Xylp residues that are partially sulfated on C-4.

It has been identified that a structurally novel fucoidan SFP, a polysaccharide fraction extracted from SF, is composed mainly of fucose and galactose in a ratio of 73.16% to 26.84%, respectively, through inverse HPLC techniques [11]. The results showed that the sulfate content was relatively high (23.6%), while the protein content was the lowest (1.21%). Methylation analysis revealed fucose residues and galactose residues, mainly in the forms of →3)-L-Fucp-(1→, →4)-L-Fucp-(1→, →3,4)-L-Fucp-(1→, →3)-D-Galp-(1→, and →3,6)-D-Galp-(1→. The Hakomori method was primarily used for methylation. Additionally, non-reducing ends such as →2,4)-L-Fucp-(1→ were also identified. The sulfation sites on fucose residues were typically located at C-4 and C-2/3. The main chain primarily consisted of alternating →3)-α-L-Fucp-(1→, →4)-α-L-Fucp-(1→, →3,4)-α-L-Fucp-(1→, and →3)-β-D-Galp-(1→ residues, with a minor portion of →6)-β-D-Galp-(1→ also present. In comparison to another study by this researcher [39], SFP was found to contain low amounts of glucose (3.66%), mannose (2.54%), and glucosamine (0.42%), in addition to fucose (66.81%) and galactose (26.57%). The residue composition of SFP remained consistent with previous findings, with fucose residues sulfated at the C-3 and C-4 positions, where the C-3 position was identified as the major site of sulfation. The structural characteristics of SFP are illustrated in Figure 2A.

SFF-32 is a fucoidan fraction isolated from SF [40]. The molar percentage of each monosaccharide determined by HPLC method was Fuc(21.5%): Man(26.9%): Rha(18.5%): GlcA(9.9%): Xyl(9.7%): Gal(7.7%): Glc(5.8%). The monosaccharide composition exhibited slight differences from those reported in previous studies, the possible reasons for this may be attributed to the different extraction and purification methods chosen, or differences in the source of Sargassum as well as the harvesting season. SFF-32 is mainly composed of sugar (64.06%), uronic acid (28.21%), and sulfate (5.43%), with no protein. The molecular weight is 47.3 kDa. Methylation analysis showed that SFF-32 has four major substations: →4)-D-Manp- (1→, →3)-L-Fucp-(1→, →4)-L-Rhap-(1→, and T-linked Xylp. The terminal Xylp can be used as a functional glycosyl modification site involved in specific binding to cellular receptors or enzymes to improve their biorecognition. The predicted structure of fucoidan is shown in Figure 2B.

F32 is a fucoidan fraction extracted from Hizikia fusiforme and purified by column chromatography. It has a molecular weight of 92.7 kDa and contains 21.8% sulfate [41]. F32 is a novel fucoidan with no fucose core. The molar percentages of Fuc, Man, Gal, Xyl, Glc, Rha, Ara, and in the fucoidan are 41.7%, 24.4%, 20.6%, 6.1%, 2.0%, 3.6%, and 1.5%, respectively. The core backbone structure of F32 is alternately linked by (1→2) linked-α-D-Man and (1→4) linked-β-D-GlcA. Approximately two-thirds of the fucose residues of F32 are located at the non-reducing end. The sulfation sites are mainly located on C-6 and C-4 of Man residues; C-3 of Gal residues; and C-2, C-3, and C-4 of fucose residues. The main fucoidan fraction, YF5, was isolated from Hizikia fusiforme using anion exchange chromatography and then depolymerized by partial acid hydrolysis. Its main chain structure was identified as an alternating repeating disaccharide unit of →2)-α-D-Man-(1→ and →4)-β-D-GlcA-(1→ using the ES/CID-MS/MS technique [42]. It is consistent with the results of the exploration of the structure of F32. The structure is effective in enhancing the interactions between organisms. This is because the fucose in the terminal position can mimic the glycans on the host cell surface and inhibit pathogens through competitive binding. Meanwhile, the excessive hydroxyl group exposure can form hydrogen bonds with water molecules and increase the hydrophilicity of F32.

As shown by previous studies, some researchers isolated a novel fucoidan (SFPS65A) from SF. The crude polysaccharide extracted from SF, SFPS65, was further fractionated and purified to obtain SFPS65A, which has a molecular weight of 90 kDa and contains 17.5% sulfate, and was found to be composed of fucose, mannose, galactose, xylose, glucose, and uronic acid with an inter-ratio of 19.23:2.57:9.58:6.64:1:6.52 after monosaccharide analysis [43]. The main chain of SFPS65A consists of alternating [→3)- β-L-Fuc p-(1→] and [→3,4)- β-L-Fuc p-(1→] units. It is important to note that the fucose in these units has an α-configuration, not β. And in another study by this researcher, it was pointed out that the fucoidan (SFPS65-B) possesses exclusively 1,4-glycosidic linkages. The main chain of the polysaccharide is composed of alternately repeating arrangements of →4)-α-GalpA-(1→, →4)-α-Hex-(1→ and →4)-α-Fucp-(1→ units [44]. The structure of the SFPS65-B is shown in Figure 2C.

Two fractions of fucoidan, F1-2 and F2-2, have been purified from crude Hizikia fusiforme polysaccharide using gel column chromatography [45]. F1-2, a fucoidan with a molecular weight of 39 kDa, consists of sugar (59.8%), uronic acid (6.0%), protein (0.8%), and sulfate (24.0%) fractions. It is composed of the following monosaccharides: Fuc (67.9%), Gal (15.8%), Xyl (8.2%), GlcA (3.7%), Man (2.6%), and Rha (1.8%). The components and monosaccharide types of F2-2 were consistent with F1-2, but there were differences in content. Its molecular mass was 50 kDa and the ratio of monosaccharide molecules was Fuc: Gal: Xyl: GlcA: Man: Rha = 68.6: 20.4: 5.9: 2.6: 1.6: 1.0. The researchers explored the structure of F2-2 by methylation combined with NMR and GC-MS analysis, and found the main chain of F2-2 is →3)-α-L-Fucp-(1→, with branches present at the C-2 or C-4 positions. The core fragment of F2-2 is shown in Figure 2D.

From the above studies, it is clear that the complex and diverse structures of fucoidan are mainly attributed to multiple factors such as monosaccharide composition, terminal group, main chain structure, molecular weight differences, and extraction methods. These structural variations affect the physicochemical properties of fucoidan, such as solubility, charge distribution and molecular conformation, which in turn determine its biological activity. For example, sulphated modifications and molecular weight significantly affect the antioxidant, antiviral and immunomodulatory activities of fucoidan by modulating interactions with biological targets, including free radicals, viral surface proteins and immune receptors [46].

### 3.2. Alginic Acid

Alginic acid, also known as alginate or kelp gum, is the sole carboxylate-containing component of the polysaccharide fraction in SF. It easily combines with various cations found in seawater to form diverse types of alginates, primarily located in the cell wall of seaweed or secreted into the outer layer of a cell. Guluronic acid, a component of alginic acid, has the ability to bind with various divalent cations, including Ca^2+^, Ba^2+^, and Sr^2+^ [47]. This interaction facilitates intermolecular cross-linking, which in turn results in the formation of hydrogels. Such properties have led to a wide range of applications for alginic acid in the food industry, cosmetics, pharmaceuticals, and biomaterials.

**Figure 3:**
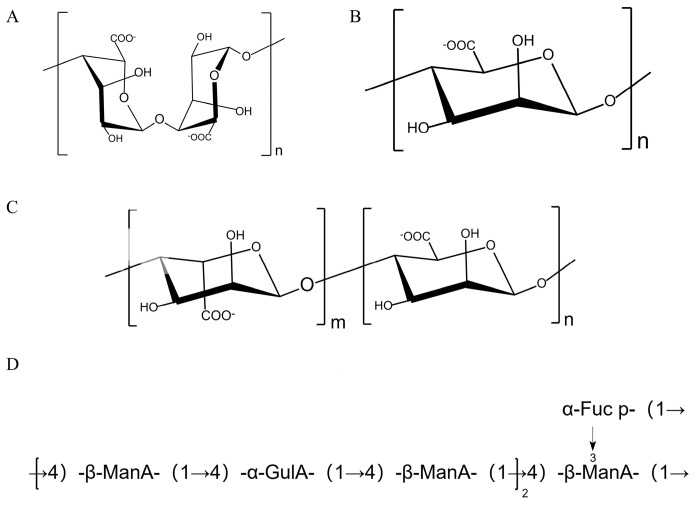
The typical structure of alginic acid. (**A**) PG, (**B**) PM, (**C**) PGM, (**D**) the prediction structure of P1.

Alginic acid is a linear polysaccharide formed by the non-repetitive, chimeric linkages of β-D-mannuronic acid and α-L-guluronic acid units, comprising polyguluronic acid (PG), polymannuronic acid (PM), and a mixed sequence of both residues (PGM) [48]. The structures of PM, PG, and PGM are shown in Figure 3. The ratio of M/G in alginic acid may vary with the type of raw material, ecological environment, determination method, and harvesting season of SF.

Fraction P1, primarily consisting of alginic acid with a molecular weight of 289 kDa, has been isolated and purified from SF [36]. It is composed mainly of mannuronic acid, guluronic acid, and a minor portion of fucose in a molar ratio of 7.67:2.35:1.00. The main chain of P1 consists of alternating repeats of →4)-β-ManA-(1→ and →4)-α-GulA-(1→ units, with the terminal group α-Fuc p-(1→ connected to →3,4)-β-ManA-(1→ via O-3 sites. The prediction structure of P1 is shown in Figure 3D. Meanwhile, researchers have found that alginate has a better anti-photoaging effect. But its corresponding mechanism remains to be further explored by researchers.

Fraction 04S2P, with a molecular weight of 29.2 kDa, has been successfully isolated and purified from SF [47]. The monosaccharide composition of 04S2P was analyzed by HPLC and it was found that the substance consisted only of mannuronic acid and glucuronic acid, with a molar ratio of (M/G) 9.0: 1.0. Another researcher isolated an alginate named SFP using DEAE-Sephadex A50 and Sephadex G-100 chromatographic techniques [49]. The alginate has a molecular weight of 16 kDa and features a molar ratio of mannuronic acid (M) to guluronic acid (G) of 2.75:1.0. Differences in M/G may also be related to the source of the material, measurement techniques, and harvest seasons, or other factors.

### 3.3. Laminaran

**Figure 4:**
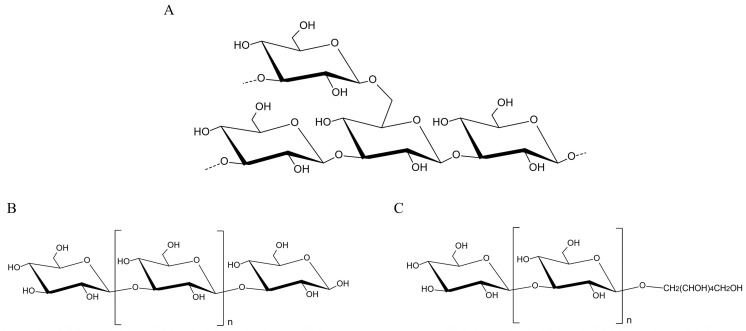
The characteristics of the structure of laminaran. (**A**) The typical structure of laminaran, (**B**) G-chain laminaran, (**C**) M-chain laminaran.

Laminaran is a type of small-molecule neutral polysaccharide that contains large amounts of β-glucose, and its molecular weight primarily depends on the number of polymerized glucose units. It was demonstrated that the chemical structure of laminaran comprises a large number of β-(1,3) and β-(1,6) glycosidic bond-linked β-D-glucose residues. The terminal 1-O position may be substituted by either mannitol or glucose residues, referred to as M-chains and G-chains, respectively [50]. Figure 4 illustrates the structural composition of laminaran. The ratio of 1-3 to 1-6 glycosidic bonds also influences the water solubility of laminaran in cold water. The polysaccharide is insoluble when the structure predominantly consists of (1,3)-β-D-glucose, but becomes soluble in water when there is a significant presence of (1,6)-β-D-glucose [51]. Laminaran possesses a multitude of biological functions, such as anti-cancer, antioxidant, immunity regulation, anti-inflammatory, and so on.

Previous studies have demonstrated that HFS-1 is a fraction obtained from the crude polysaccharide (HFS) of Hizikia fusiforme by extraction with 0.1 M HCl, followed by anion exchange chromatography. It has a molecular weight of 3.6 kDa and consists solely of glucose [52]. Researchers have analyzed its chemical structure and revealed that HSF-1 contains 1,3-β-D-glucose and 1,6-β-D-glucose, and terminates with glucose residues. After methylation analysis, it was determined that laminaran contained 3-linked β-D-glucopyranose, non-reducing terminal glucose, 3,6-linked β-D-glucopyranose, and 6-linked β-D-glucopyranose, with the respective molar ratios of 66.4%, 15.3%, 14.2%, and 4.1%.

## 4. Extraction, Isolation, and Purification of Polysaccharides from SF

SFPs are rich in bioactivities and have great potential for applications in food, pharmaceutical, and cosmetic applications. Their extraction, isolation, and purification are essential to obtain bioactive components while maintaining their structural integrity. Notably, the choice of extraction method significantly influences both the complexity of subsequent purification steps and the quality of the extracted polysaccharides, as it directly affects purity, molecular weight distribution, and the extent of impurities such as proteins, pigments, and salts present in the final product. Therefore, selecting an appropriate extraction, isolation, and purification process is crucial for maximizing the yield of the target polysaccharides and minimizing impurity interference in the final product. Naturally, this outcome still depends on the specific experimental conditions and environment.

### 4.1. Extraction

The extraction of polysaccharides usually begins with the selection and preparation of high-quality Sargassum. The polysaccharides were initially extracted through a variety of methods, including hot-water extraction, alkaline extraction, acid extraction, enzymatic digestion, ultrasound-assisted extraction, and microwave-assisted extraction. To improve the yield and quality of polysaccharides, appropriate methods can be selected and applied individually or in combination based on the experimental objectives. In addition to the traditional extraction methods, many innovative extraction techniques have been applied to the extraction of seaweed polysaccharides in recent years. The following are some new or improved extraction techniques: high-pressure extraction, pulsed electric field extraction, supercritical fluid extraction, microbial fermentation, and so on. These methods can be employed to manipulate the yield, purity, molecular weight, and molecular structure of polysaccharides, thereby influencing the biological activity of the extracts. Different extraction methods possess their unique merits and should be selected based on the specific experimental conditions. The different extraction methods of SFPs are shown in Table 2.

#### 4.1.1. Hot-Water Extraction

Hot-water extraction is a widely used and traditional method for isolating seaweed polysaccharides. Despite the mild experimental conditions which can effectively protect structural integrity, the extraction process is lengthy and inefficient. The general procedure for extracting polysaccharides from dried SF includes crushing the dried seaweed and extracting it with hot distilled water at a solid–liquid ratio of 1:10 to 1:30 for approximately 3 h at a controlled temperature of 80 to 100 °C. Meanwhile, the extraction pH is maintained around 7 to avoid degradation of polysaccharides. The polysaccharides derived from this method contain a significant amount of co-extracted impurities, including proteins and pigments, which complicate the subsequent isolation and purification processes [59]. In a previous study, the researcher utilized the hot-water extraction method by placing dried and defatted seaweed at 80 °C for approximately 3 h, using a solid–liquid ratio of 1:30 [13]. WSFF is a prepared fucoidan extracted from SF by using a hot-water extraction method. The yield of WSFF collected by hot-water extraction was only 4.63% with a molecular weight of 65.34 kDa. The lower molecular weight of WSFF is attributed to the subsequent series of isolation and purification steps it undergoes, including DEAE–cellulose anion exchange chromatography, the Sevag method, and dialysis. Its monosaccharide composition was significantly different from the polysaccharides extracted by the other two methods. And it was also found that fucoidan extracted by hot water retained the helix-like conformation which is essential for immunological activity. This may be due to the fact that this method avoids tertiary structure damage to polysaccharides such as strong acids or alkalis. At a concentration of 800 μg/mL, WSFF demonstrated a significantly higher DPPH (83.3%) and hydroxyl-free radicals (80.9%) scavenging activities compared to the other two groups, suggesting that WSFF has a strong antioxidant effect. The likely reason for this is that WSFF contains high levels of uronic acid, which has been previously reported to be a key factor in regulating antioxidant activity [60].

These findings emphasize the complex relationship between extraction conditions, polysaccharide structure and biological activity. However, the low efficiency and high impurity content of the hot-water extraction method limit its scalability and practical application. Future research should focus on optimizing the extraction parameters or combining hot water extraction with other advanced techniques that could further improve the extraction efficiency while preserving the bioactive structural features.

#### 4.1.2. Acid Extraction

Acid extraction is commonly used to extract specific polysaccharide fractions, e.g., the release of some acid-soluble polysaccharides (e.g., fucoidan) can be facilitated by adjusting the acidic environment [61]. Acid extraction is performed by mixing finely chopped dried SF with an acid solution and placing the mixture in a 70 °C water bath for a period ranging from 1 to 4 h. Commonly, 0.1 to 1 M hydrochloric acid (HCl) is used, with a solid–liquid ratio typically ranging from 1:20 to 1:50, which can be specifically adjusted based on the scale of the experiment. Notably, while higher acid concentrations and extraction temperatures can enhance the release of polysaccharides, they also pose a risk of causing their degradation. As shown in previous studies, SFP-3-40 is a fraction of SFP obtained by acid extraction at pH 3, followed by graded precipitation with 40% (*v*/*v*) ethanol solution. When dried SF powders were treated with hydrochloric acid (pH 3), distilled water, and alkaline solutions, the resulting polysaccharide fraction SFP-3-40 exhibited a high total sugar content (74.74%) and a relatively low molecular weight (46.98 kDa). Additionally, it contained 0.03% protein, 41.43% uronic acid, and 8% sulfate [62]. Due to its low molecular weight, the polysaccharide is more conducive to digestion, absorption, and bioavailability. Consequently, SFP-3-40 binds more readily to molecules in the body, thereby enhancing its antioxidant activity, immune response, and anti-tumor activities. The structural and rheological analyses of SFP-3 provide further insights into the effects of acid extraction. Upon morphological analysis, the surface of SFP-3 was found to be relatively loose and distorted, suggesting that the polysaccharide may be partially degraded. Furthermore, rheological analysis revealed that the polysaccharide extracted under acidic conditions exhibited the lowest viscosity when precipitated at a certain ethanol concentration. This is primarily because small-molecule polysaccharides do not easily overlap and have weak intermolecular interaction forces.

#### 4.1.3. Alkali Extraction

Alkali extraction is commonly used to extract alkali-soluble polysaccharide fractions such as fucoidan and other polysaccharides containing sulfate groups [63]. The alkali extraction method is characterized by destroying the cell wall of algae through alkali treatment, releasing the tightly bound polysaccharide components. Alkaline extraction is carried out by dissolving seaweed powder in NaOH (pH = 10) for 2–4 h. The collected extract needs to be neutralized with dilute hydrochloric acid to prevent the alkaline degradation of the polysaccharides, thereby minimizing the breakage of sugar chains under alkaline conditions. In one study, researchers dissolved Sargassum in a 3% Na_2_CO_3_ solution and grouped them according to the alkali: algae ratio to test the properties related to sodium alginate [64]. The results of the study point to the fact that increasing the proportion of alkaline solution can increase alginate production under high-temperature conditions. At the same time, researchers also found that increasing the alkali–algae ratio and reaction time could alter the structural composition of alginate, thereby resulting in a higher M/G ratio. This may indicate that the glycosidic bonds between guluronic acid are more easily broken during alkaline extraction. High M/G ratios exhibit strong antioxidant activity, likely because the α-1,4 glycosidic linkages in the G blocks may decrease the ability to provide atoms [65]. Additionally, its ferric-reducing ability also enhanced with increasing pH. Therefore, it was concluded in this study that an alkaline pH enhanced the antioxidant activity of sodium alginate. However, it is important to note that an excessively high pH can degrade the polysaccharide.

#### 4.1.4. Ultrasonic-Assisted Extraction

The ultrasonic-assisted extraction method is a kind of extraction method using ultrasonic cavitation effect, thermal effect, and mechanical vibration to accelerate the penetration of a solvent and promote cell wall breaking and polysaccharide release [66]. Compared to conventional methods, ultrasonic extraction can shorten extraction time, reduce costs, and increase efficiency while minimizing thermal degradation. Therefore, it is a very practical technology at the industrial level. The steps of the ultrasonic extraction method are as follows: the pre-treated seaweed powder is mixed with the extraction solution into an ultrasonic processor, and the ultrasonic treatment is carried out according to the preset power and time. During the treatment process, the temperature is monitored at regular intervals. If necessary, the ultrasonic power is adjusted, or cooling measures are taken to ensure that the temperature remains within the set range. Samples of the algal polysaccharides were subsequently obtained through a series of centrifugation, concentration, ethanol precipitation, and lyophilization. In a study of the ultrasonic-assisted extraction of SFPs, the researchers found that under optimal reaction conditions (ultrasonic power 200 W, ultrasonic time 15 min, material-to-liquid ratio 1:50 (*w*/*v*), and water bath time 130 min), crude SFP was extracted at 25.8% [67]. In fact, the extraction method used in this study resulted in a yield of SFP that was approximately 10 percent higher compared to the experiments using the conventional hot-water extraction method [68]. In addition, research has also indicated that SFP has a strong scavenging capacity for DPPH (2,2-Diphenyl-1-picrylhydrazyl), HO radicals (Hydroxyl radicals), and ABST radicals (2,2′-Azinobis-(3-ethylbenzothiazoline-6-sulfonic acid) radicals). The reason is that ultrasound may cause the breakdown of polysaccharide long chains into smaller structures, thereby enhancing their biological activity. In another study, the yield of SFPs extracted by ultrasound-assisted extraction was 2.22% higher than that of hot-water extraction [69]. Meanwhile, the molecular weight and structure of polysaccharides were changed. In this experiment, SFPs exhibited a stronger DPPH free radical scavenging ability compared to extracts from other methods, attributed to the varying degrees of degradation induced by ultrasound. This finding is consistent with the results from the preceding study.

#### 4.1.5. Microwave Extraction

Microwave extraction, which is characterized by high yield and short reaction times, is a technique that utilizes the rapid heating effect of microwave radiation to promote cell breakage and enhance polysaccharide solubilization and release. Its polysaccharide extracts usually have a high sulfate content and low molecular weight [70]. This method may change the structure of polysaccharides, which in turn affects their physicochemical properties and biological activities. Microwave treatment has been reported to disrupt the crystalline structure of polysaccharides and roughen the surface of polysaccharides, which is conducive to increasing their reactivity. It also triggers the breakage of polysaccharide molecular chains and modifies the chemical structure, which in turn affects the spatial structure and function of the polysaccharides [57]. As shown by previous research, researchers mixed seaweed powder with an extraction solvent and subjected the mixture to microwave treatment at a solid–liquid ratio of 1:25, a power of 560 W, and a duration of 1 min [71]. The results showed that the products obtained through microwave-assisted extraction have lower molecular weights than those obtained through other traditional extraction methods. It was also observed that fucoidan extracted using the microwave-assisted extraction method exhibited a higher toxicity to adenocarcinoma cells. This may be attributed to the presence of a galactose backbone in fucoidan, which facilitates the efficient binding of fucoidan to glycoproteins or receptors on the surface of cancer cells. Another study also showed that the crude extract from the microwave-assisted extraction method had strong biological properties with significant antiproliferative and antioxidant effects [72].

#### 4.1.6. Enzyme Extraction

Enzyme extraction employs the catalytic action of specific enzymes to degrade the cell walls of algae, which in turn releases the polysaccharide components. Commonly used enzyme preparations for extraction include cellulase and hemicellulase (mainly used to destroy cellulose and hemicellulose structures in the cell wall), pectinase (used to degrade pectin and facilitate the release of polysaccharides), and protease (used to degrade protein components in the cell wall). The general steps of the enzyme extraction method are as follows: freeze-dried seaweed powder is mixed with distilled water or other reagents, stirred well and added with an appropriate amount of enzyme preparation. The enzymatic reaction is usually carried out at 50 °C and a pH of approximately 4.5. After the enzymatic digestion is completed, the enzyme preparation is inactivated by heating or pH adjustment to prevent the enzymatic degradation of polysaccharides in subsequent reactions. Different enzymes exhibit varying efficiencies in polysaccharide extraction. Given the cell wall composition of SF, the appropriate selection of enzyme combinations can markedly enhance extraction efficiency. For instance, a blend of cellulase and pectinase is commonly utilized to break down the cell wall [73]. Researchers utilized Celluclast, a cellulase enzyme, to enzymatically dissolve freeze-dried seaweeds at pH = 4.5 and a temperature of 50 °C for 24 h [74]. The amount of Celluclast added was about 5% of the seaweed. After digestion, the enzyme was inactivated by heating at 100 °C for 10 min. Finally, the polysaccharides of Hizikia fusiforme (HFPS) were obtained by ethanol precipitation. The yield of HFPS was 29.35%; compared with the traditional chemical extraction method, the enzyme extraction method was effective in improving the extraction rate of polysaccharide. Researchers discovered that HFPS extracted by the enzymatic method, rich in fucose, galactose, and sulfate, demonstrated potent antioxidant activity in both in in vivo and in vitro experiments. The probable reason for this is that the enzymatic method selectively cleaves the cross-linking sites between polysaccharides, thereby exposing more functional groups, such as sulfate and hydroxyl groups.

#### 4.1.7. Other Extraction Methods

In addition to traditional extraction methods such as hot-water extraction, alkali extraction, acid extraction, and ultrasound-assisted extraction, numerous innovative extraction techniques have been applied for the extraction of seaweed polysaccharides in recent years.

It has been reported that the polysaccharides of SF were extracted via hydrogen peroxide/ascorbic acid-assisted extraction by mixing the seaweed powder with distilled water at 90 °C for 2.5 h [30]. The hydrogen peroxide/ascorbic acid solution was then added to the solution at 50 °C and heated for 30 min. This method showed the highest yield (11.43%) and relatively high bioactivity of polysaccharide (SFP) compared to the other five conventional methods.

Besides hydrogen peroxide/ascorbic acid-assisted extraction, several other novel green extraction technologies have been developed for polysaccharide isolation. Ultra-high-pressure extraction of polysaccharides is a new type of green extraction technology [75]. By applying pressure higher than atmospheric pressure, the permeability of the cell wall can be changed and the structure of the plant cell can be damaged, which promotes the solubilization of polysaccharides in the cell and improves the extraction efficiency. The pulsed electric field extraction technique is a technique in which samples are treated with short-duration high-voltage pulses to cause changes in the potential gradient of the cell membrane, thereby increasing the permeability of the cell membrane and promoting the release of polysaccharides and other substances [76].

Another promising method is supercritical fluid extraction, which utilizes CO_2_ (or other fluids) in a supercritical state as a solvent. Due to its gas-like diffusivity and liquid-like solubility, supercritical CO_2_ can efficiently extract a broad spectrum of bioactive substances [77]. Moreover, microbial fermentation extraction has recently been proposed, in which different microorganisms are selected to target specific polysaccharides. Notably, these microbes can also modify the chemical structure of polysaccharides, potentially altering their biological activities [78].

### 4.2. Isolation and Purification

Isolation and purification are key steps in polysaccharide research, aiming to remove proteins, pigments, salts and other small-molecule impurities to obtain highly pure polysaccharide samples for structural elucidation and biological function studies. The structural properties of polysaccharides, such as molecular weight, sulfate group content, and uronic acid ratios, are often influenced by purification conditions, thereby affecting their biological functions, including antioxidant, antitumor, immunomodulatory activities, and so on. Reasonable isolation and purification strategies can not only enhance the purity of polysaccharides, but also effectively protect the key functional groups and their structural integrity to avoid triggering molecular degradation or the loss of function during the purification process.

#### 4.2.1. Removal of Impurities

The key role of deproteinization in polysaccharide isolation and purification is not only reflected in the increase in purity and improvement of functionality, but also provides new ideas for the precise characterization and application exploration of polysaccharides. By employing advanced deproteinization techniques, a significant reduction in protein interference can be achieved, enhancing the sensitivity of analytical and detection methods, and thereby facilitating the precise structural analysis and functional screening of polysaccharides. At the same time, it prevents interference with the biological activity and safety of polysaccharides, and avoids allergenicity or false-positive test results due to protein residues. This removal is typically achieved through several methods, including the Sevag method, which involves repeated extraction with a chloroform/n-butanol (4:1, *v*/*v*) mixture to efficiently remove protein [79], macroporous resin adsorption [80], the trichloroacetic acid (TCA) method [81], and freeze–thaw treatment [82]. The Sevag method has been widely used due to its advantages, including minimal damage to the polysaccharide backbone and active groups, as well as its broad applicability [83]. However, the method also faces drawbacks, such as the toxicity of the solvents used, the time-consuming nature of the process, and the fact that it can only remove a small amount of protein at a time, making it inefficient. In the future, the Sevag method should be continuously optimized under the concept of green chemistry to improve efficiency while reducing environmental pollution. Macroporous resin adsorption is favored for its strong adsorption capacity, high selectivity, and reusability [84]. In recent years, it has thus become widely used as an adsorbent in various fields, including environmental protection, industrial decolorization, and the separation and purification of active ingredients in natural products. It is worth mentioning that although macroporous resin adsorption can efficiently adsorb and remove protein impurities from solution, the removal of certain proteins may be limited by the type of resin, especially for proteins with molecular weights close to polysaccharides or with complex structures. And there may be strong interactions with the resin, leading to elution difficulties. The TCA method is simple and efficient. However, TCA is a strong irritant and toxicant, posing risks to laboratory personnel and the environment. Moreover, it can cause partial hydrolysis of the target polysaccharide, leading to variable biological activity [85]. It has been reported that some researchers found that the TCA method has a better deproteinization of polysaccharides (95.3%), but it has a higher percentage of polysaccharide loss (18.9%) [86]. Freeze–thaw treatment for protein removal is a non-polluting, low-cost, and simple physical method, but the removal effect is limited and time-consuming.

Initially extracted crude polysaccharides may also contain many pigments, inorganic salts, and other small-molecule impurities. Researchers commonly employ activated carbon adsorption and hydrogen peroxide to eliminate pigments and utilize dialysis techniques to remove salts and other low-molecular-weight impurities, thereby achieving the isolation of polysaccharides with an enhanced purity. Regarding decolorization, the application of various decolorization methods exerts distinct influences on the structural composition and bioactivity of polysaccharides. There are studies that have reported that activated-carbon-treated samples have higher molecular weights (306.4 kDa) relative to hydrogen peroxide, and have more sulphate groups (14.33%, *w*/*w*). However, samples treated with hydrogen peroxide exhibit high levels of uronic acid (35.02%) [87].

#### 4.2.2. Chromatography Column Separation

The main objective of performing chromatography column separations is to accurately separate and purify the target polysaccharide fractions for subsequent research or applications. Chromatography column separation is one of the most accurate polysaccharide purification steps available, and is particularly suitable when high purity, multi-stage separations are required. It typically includes gel filtration chromatography and anion exchange chromatography. Given that SFPs are acidic polysaccharides, researchers have utilized the charge characteristics of polysaccharides to separate them based on the binding strength of the acidic groups of the polysaccharides to the charged groups on the filler, typically using the DEAE-Sepharose series, DEAE-Cellulose anion exchange, and DEAE-52 anion exchange. Separation is then carried out by molecular weight using the Sephadex, Sephacryl or Superdex series of separation methods. For example, a water-soluble polysaccharide (SFPS) was isolated and purified from SF, which has a molecular weight of 299 kDa, first using DEAE-52 cellulose anion exchange and then Sephadex G-200 gel filtration chromatography [34].

## 5. Biological Functions of Polysaccharides from SF

### 5.1. Antioxidant Effect

Polysaccharides from SF have received widespread attention for their remarkable antioxidant properties. Their antioxidant efficacy is mainly achieved through free radical scavenging, metal chelation, lipid peroxidation inhibition, and an enhancement of the endogenous antioxidant system.

SFPs, which are extracted from SF using water extraction and alcohol precipitation, exhibits strong scavenging ability due to its special molecular structure, particularly its sulfate group (SO_4_^2−^) and hydroxyl groups, which possess powerful free radical scavenging activity [68]. It has also been shown that SFPs are able to protect cells from oxidative damage by reacting with these free radicals and reducing the generation of ROS in the cell [88]. Furthermore, researchers have discovered that H-SFP, a polysaccharide from SF using hot-water extraction, demonstrated stronger 1,1-diphenyl-2-picrylhydrazine (DPPH), hydroxyl radical, and ABTS radical scavenging ability when tested for antioxidant activity in vitro. The two fractions eluted with 0.3 mol/L NaCl and 0.5 mol/L NaCl were named H-SFP3 and H-SFP5, respectively [30]. H-SFP scavenged DPPH radicals up to 95.35% at a concentration of 4 mg/mL, which was close to the scavenging ability of vitamin C (often used as a standard antioxidant). Moreover, within the concentration range of 0.5–5 mg/mL, the scavenging rate of DPPH radicals was positively correlated with the concentration of polysaccharides. One of the reasons for this may be that the sulfate group is able to provide hydrogen atoms, which then react with the DPPH radical, thereby causing the radical to be reduced. The IC50 values of hydroxyl radical scavenging by H-SFP ranged from 2.03 to 2.45 mg/mL, which was significantly better than the antioxidant capacity of other natural polysaccharides. The scavenging ability of hydroxyl radicals is closely related to the uronic acid content of H-SFP, which has a superior metal chelating ability and can effectively chelate Fe^2+^ in the Fenton reaction to inhibit hydroxyl radical generation [33]. At a concentration of 5 mg/mL, the scavenging rate of ABTS radicals by H-SFP was 64.78%, which was significantly higher than that of H-SFP3 and H-SFP5, the later of which were 39.66% and 38.85%, respectively. The difference in this scavenging capacity stems from the molecular weight and degree of sulfation of the H-SFP. H-SFP with a higher content of sulfate groups demonstrate stronger ABTS radical scavenging capacity. Meanwhile, polysaccharides with smaller molecular weights are able to react with free radicals more rapidly due to their higher diffusivity. H-SFP also exhibited significant antioxidant protection at the cellular level. In the hydrogen peroxide (H_2_O_2_)-induced oxidative stress model of RAW264.7 macrophages, H-SFP effectively inhibited the rise of intracellular ROS levels. Additionally, H-SFP was able to significantly increase intracellular superoxide dismutase (SOD) enzyme activity in a concentration-dependent manner. Among the various polysaccharide species tested, H-SFP3 had the strongest effect, with a 145.67% increase in SOD activity.

The antioxidant activity of polysaccharides extracted by different methods from SF was compared [13]. The hot-water-extracted polysaccharide (WSFF) demonstrated a hydroxyl radical scavenging rate of 80.9% and a DPPH radical scavenging rate of up to 83.3% at the concentration of 800 μg/mL. In contrast, the polysaccharide extracted using acid (ASFF) showed significantly lower scavenging rates for both DPPH radicals (28.1%) and hydroxyl radicals (26.9%). It has been found that higher-molecular-weight polysaccharides typically exhibit superior antioxidant capacity due to their more complex molecular structures. This complexity enhances their ability to scavenge free radicals. The findings indicated that WSFF demonstrated potent antioxidant efficacy, particularly in scavenging DPPH and hydroxyl radicals. This is attributed to its high content of sulfate groups and uronic acid. The antioxidant capacity of WSFF is closely related to the extraction method, molecular weight, structure, and functional group content. It has been pointed out that SFP was able to significantly increase the levels of SOD and glutathione (GSH) in mice [89]. These enzymes serve as the primary defense mechanism against oxidative stress in the body, mitigating free radical-induced damage by scavenging ROS. In a cyclophosphamide (CY)-induced immunosuppression model, SFP significantly reduced malondialdehyde (MDA) levels in mouse livers, suggesting its potential to attenuate oxidative stress-induced lipid peroxidation damage. The DPPH radical, hydroxyl radical and superoxide anion radical scavenging ability of carboxymethylated and hydroxamated modified *Sargassum fusiforme* polysaccharide (HCDPSF) was significantly enhanced compared to conventional polysaccharides [21]. Meanwhile, it is noteworthy that HCDPSF, particularly in the HCDPSF-2 condition (which utilized 3.0 mmol of EDC and 3.0 mmol of hydroxylamine hydrochloride), exhibited a stronger iron-chelating ability. Its capacity to reduce ferric ions approached 95% that of vitamin C, the property that may be attributed to the introduction of hydroxamic acid, which improves the chelating ability.

SFPs exhibit potent antioxidant efficacy via multiple mechanisms, such as free radical scavenging, enhancement of antioxidant enzyme activity, and inhibition of oxidative stress. Their molecular weight, composition, chemical modification and extraction method significantly affect their antioxidant activity. As a natural and non-toxic antioxidant, SFPs hold a broad spectrum of potential applications, particularly in the realms of functional foods and pharmaceuticals.

### 5.2. Anti-Tumor Activity

SFP fractions from SF have shown significant antitumor effects both in vivo and in vitro. Numerous studies have demonstrated that specific polysaccharide fractions from SF, such as SFPs, can inhibit tumor cell proliferation and induce apoptosis.

A nasopharyngeal carcinoma model has been successfully established in mice through the inoculation of CNE cells, as per previous research [90]. Following intervention with *Sargassum fusiforme* polysaccharide (SFP), they observed a sequential decrease in tumor weight from the control group to the high-dose group. Notably, the tumor inhibition rate in the 200 mg/kg group reached as high as 63.3%, and the inhibition effect of SFPS on the growth of tumors was dose-dependent. Pathological sections of the tumor tissue revealed that the structure of the tumor tissue had suffered severe damage following SFP treatment, characterized by extensive necrosis and a significant reduction in tumor vascularity. The serum levels of TNF-α, IL-1β, NO, and IgM in mice, which are key modulators of immune and inflammatory responses, were found to be significantly elevated. In addition, SFPs may enhance immune responses by activating TLR receptors and the p38 MAPK signaling pathway. In another of the researchers’ in vivo and in vitro experiments on SFPs resistance to liver cancer, it was noted that the effect of SFPS on inhibiting the proliferation of HepG2 cells was more significant with increasing concentration and time, as detected by CCK-8. And at the highest concentration of 2000 μg/mL intervention, the inhibition rate of HepG2 cells reached 90%. In vivo experiments were performed by inoculating tumor cells into mice to establish a liver cancer model. SFP was administered to the mice at different doses (100 mg/kg, 200 mg/kg, and 400 mg/kg), and the results showed that the tumor inhibition rate increased with the dose. Additionally, the spleen weight, serum levels of cytokines (such as IL-1, TNF-α and NO), and IgM secretion levels of the mice were also significantly elevated. Flow cytometry analysis indicated that SFP was effective in inducing apoptosis in tumor cells within a certain range. Further exploration revealed that the pro-apoptotic mechanism of SFP may be due to the fact that it can regulate Bcl-2 family proteins and inhibit the anti-apoptotic protein Bcl-2, thus enhancing the expression of the pro-apoptotic protein Bax. It has been reported that SFP also demonstrates significant anti-tumor effects on lung cancer cells via multiple mechanisms, such as the direct inhibition of tumor cell proliferation, the suppression of tumor angiogenesis, and the modulation of the VEGF signaling pathway [91]. In in vitro experiments, SFP was able to inhibit the proliferation of SPC-A-1 cells and human umbilical vein endothelial cells (HUVECs) in a dose-dependent manner, and induced their cell cycle arrest in the G2/M phase. In vivo experiments demonstrated that SFP significantly reduced tumor volume and weight, and down-regulated the density of tumor microvessels (MVDs), as well as the expression of markers related to angiogenesis, such as CD31 and VEGF-A. The VEGF/VEGFR2 signaling pathway provides tumor cells with the necessary blood supply to support their growth and metastasis, as well as promotes tumor progression through a variety of mechanisms, including influencing the immune microenvironment. SFP also inhibited the expression of VEGFR2 in HUVECs, demonstrating similar effects even in the absence of tumor-related signals in the environment. This suggests that SFP not only directly inhibits angiogenesis, but also indirectly suppresses tumor growth by modulating the VEGF signaling pathway.

### 5.3. Immunomodulatory Effects

The immunomodulatory effects of polysaccharide fractions from SF are primarily mediated by the activation of macrophages and modulation of cytokine activities to maintain immune homeostasis. Extensive research has confirmed that specific fractions, such as the F2 fraction and the SFP-αII fraction, enhance immune system function. A study has compared the effects of F2, a polysaccharide fraction from SF, on macrophage RAW264.7 cells at concentrations ranging from 10 to 50 μg/mL [92]. They found that the F2 fraction exhibited the strongest proliferative effect on macrophages at the highest concentration of 50 μg/mL, and induced a concentration-dependent increase in NO production. This increase was comparable to the levels induced by lipopolysaccharide (LPS). At the molecular level, F2 polysaccharide was able to promote NO production by upregulating the mRNA expression of inducible nitric oxide synthase (iNOS). The F2 fraction was also able to induce the secretion of pro-inflammatory cytokines such as tumor necrosis factor-α (TNF-α), interleukin-1β (IL-1β), interleukin-6 (IL-6), and interleukin-12 (IL-12), while stimulating the secretion of the anti-inflammatory cytokine interleukin-10 (IL-10). Western blot analysis revealed that the F2 fraction was able to induce the phosphorylation of NF-κB, ERK, JNK and p38 proteins in macrophages, suggesting that these signaling pathways are involved in the activation process of polysaccharides to macrophages. In another study where researchers used the mouse model of cyclophosphamide (CTX)-induced immunosuppression, it was found that the spleen and thymus indices of the mice were significantly decreased, indicating impaired immune function [93]. SFP-αII treatment significantly restored the weight of these organs, suggesting that SFP-αII was effective in counteracting the immunosuppression caused by CTX and enhancing the immune function. SFP-αII also enhanced the phagocytosis of macrophages and increased NO secretion. SFP-αII in the high-dose group (400 mg/kg) promoted the secretion of TNF-α and IL-1β, which play key roles in resisting infection and modulating inflammatory responses. SFP-αII promotes the phosphorylation of ERK, JNK, and p38 in the MAPK pathway by binding to Toll-like receptor 4 (TLR4) and also facilitates the phosphorylation of inhibitor kappa Bα (IκBα) and its upstream kinase, inhibitor kappa B kinase (IKK), thereby activating the NF-κB signaling pathway. This activation subsequently enhances the immunomodulatory capacity of macrophages.

### 5.4. Antiviral Activity

Polysaccharide fractions from SF also exhibit inhibitory effects on various viruses, such as respiratory syncytial virus RSV [94], avian leukemia virus type J ALV-J [95], and norovirus NoV [96]. They can exert antiviral effects by preventing viral binding to host cells, interfering with various stages of viral replication, and enhancing the immune system. Researchers used a variety of isolated and purified polysaccharides for antiviral activity screening, and polysaccharide SFP4 had the highest therapeutic index (TI > 139) with a half effect concentration (EC50) of 18.0 μg/mL, which provided the strongest activity against RSV. In the intervention with SFP4 on RSV-infected Hep-2 and A549 cells, it was observed that SFP4 exerted inhibitory effects during the preventive, viral adsorption blocking, and therapeutic phases. Among these, the preventive effect was the most pronounced, followed by the blocking of viral adsorption, with the therapeutic effect being the least significant. The reason may be that SFP4 blocks the binding of RSV viral proteins to host cell surface receptors, inhibiting viral adsorption and infection. The experiments also confirmed that SFP4 at a high concentration (500 μg/mL) had a significant inhibitory effect on the expression of F, G, and N proteins of RSV, especially on the expression of RSV-N protein, which showed concentration-dependent inhibitory effect. In heparin competition assays, SFP4 may inhibit viral binding to cellular receptors by competitively binding to them, thereby preventing viral attachment. Specifically SFP4 may have the effect of inhibiting viral infection by binding to the RSV-G protein, thereby blocking the binding of RSV to the heparan sulfate (HS) receptor.

### 5.5. Regulation of Intestinal Flora

The effect of SF polysaccharides on intestinal flora has gradually become a research focus. SFP fraction exhibits significant prebiotic properties that modulate the composition and function of the intestinal microbiota, thereby positively affecting host health. SFP also promotes the growth of beneficial flora such as bifidobacteria and lactobacilli. These beneficial floras maintain intestinal health, enhance immune function, and combat harmful flora. Based on the dilution curves and the Shannon index, it has been demonstrated that while the abundance of the flora did not significantly differ among the groups, the diversity of the flora was higher in the SFP-treated group [18]. This suggests that SFP may have played a role in promoting a more balanced and diversified development of the intestinal flora structure. A change in the relative abundance of intestinal flora was also observed in the SFP group, characterized by a relative decrease in Bacteroidetes and Proteobacteria, and a relative increase in Firmicutes and Actinobacteria. SFP also promotes the growth of bacteria that produce short-chain fatty acids (SCFAs), such as Faecalibacterium, which are crucial for maintaining intestinal health, enhancing immune function and preventing inflammation. Observations on the effects of SFP on the intestinal flora of mice fed a high-fat diet yielded results similar to those of earlier research [31]. Both concluded that SFP was more effective in improving intestinal flora.

### 5.6. Antidiabetic and Complication Efficacy

Diabetes mellitus is a chronic metabolic disease caused by insufficient insulin secretion or insulin resistance, which is mainly characterized by abnormally high blood glucose levels, and according to the division, diabetes mellitus can be classified as type I, type II, gestational or other types. SFP fraction can ameliorate the pathogenesis of diabetes mellitus through a variety of mechanisms, such as increasing insulin sensitivity, stimulating insulin secretion from pancreatic β-cells, promoting glucose uptake and metabolism, and improving the efficiency of glucose metabolism. Given the presence of strong antioxidant properties and anti-inflammatory efficacy of sargassum polysaccharides, SFP may also maintain the normal function of pancreatic β-cells by protecting them from damage due to oxidative stress and inflammation. Meanwhile, abnormalities in intestinal flora and lipid metabolism can indirectly reflect changes in the glucose level of the body. When comparing the effects of acarbose (ACAR) and SFP-ACAR combination on fasting blood glucose (FBG) and postprandial glucose in diabetic rats, it has been found that the SFP combination treatment resulted in a greater reduction in blood glucose compared to the ACAR group, with fasting blood glucose decreasing by 33.01% [97]. Meanwhile, the insulin resistance index (HOMA-IR) was significantly reduced by 63.97% in the SFP combination therapy group, compared to a reduction of only 38.63% in the group treated with ACAR alone. This indicates that SFP is more effective in reducing insulin resistance. SFP stimulates the expression levels of insulin receptor substrate-1 (IRS-1), phosphatidylinositol 3-kinase (PI3K), and protein kinase B (Akt), among others, resulting in improved insulin sensitivity. In addition, hepatic gluconeogenesis is a key factor contributing to elevated blood glucose, and SFP can effectively inhibit the expression of the key gluconeogenic enzymes phosphoenolpyruvate carboxykinase (PEPCK) and glucose-6-phosphatase (G6Pase), thereby reducing hepatic glucose output. SFP is also effective in a range of complications caused by diabetes, such as cardiovascular disease [98], and liver and kidney damage [99]. In terms of cardiovascular disease risk reduction, SFP significantly lowers total cholesterol (TC), triglycerides (TG), and low-density lipoprotein cholesterol (LDL-C), while increasing high-density lipoprotein cholesterol (HDL-C) [100]. The polysaccharide also improves the levels of liver function indicators (such as ALT, AST, ALP) and markers of renal impairment (BUN), which reduces liver injury, and protects liver and kidney function.

### 5.7. Additional Biological Effects

In addition to the common biological effects mentioned earlier, SFP fractions exhibit a range of additional benefits, including anticoagulant and antithrombotic effects [35], the promotion of bone health [101], repair of injuries resulting from over-exercise [102], and neuroprotective effects [103]. For example, SFP-1, a type of SFP, can regulate the growth factors of bone cells, stimulate the growth of osteoblasts, reduce bone loss, and protect bones from damage. To summarize, there are many evidences showing that SFPs have rich therapeutic effects, which can be further explored in the future, and applied in clinical treatment and functional food development.

**Figure 5:**
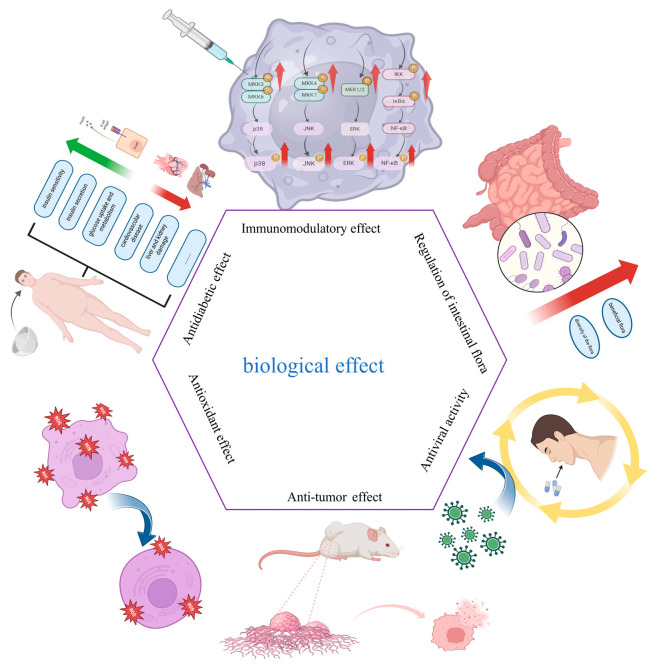
Biological effects of polysaccharides from SF.

There is a strong association between the multiple biological activities of SFPs. For example, multiple activities of SFPs modulate this association through similar signaling pathways. By activating the NF-κB pathway, this enhances the immune response of the body and its antiviral and antitumor activities. The SFP fraction also can improve glycolipid metabolism via the gut–metabolic axis [104]. Thus, this interconnectedness highlights the vast potential of SFPs in the development of functional foods and pharmaceuticals. The specific SFP biological efficacy is shown in Figure 5.

## 6. Conclusions and Prospects

SFPs are a category of natural marine polysaccharides with diverse bioactivities. The unique structural features and rich chemical compositions, as well as varying degrees of sulfate-group modifications, determine their remarkable activities in antioxidant, antitumor, immunomodulatory, and antiviral areas. In terms of structure, the molecular weight, degree of sulfation, sugar chain branching structure, and other modifying groups of SFPs have been initially shown to regulate their biological activity. This interconnectedness highlights the vast potential of these polysaccharides in the development of functional foods and pharmaceuticals. However, more advanced techniques are still needed to explore the detailed structural characterization of polysaccharides, particularly the conformational changes of sugar chains, the types of linkage groups, and the mechanisms of interaction with target tissues in vivo. For example, the structure of fucoidan is the most complex among the polysaccharide components, but its main structure consists of L-fucose interconnected by α-(1→3) or α-(1→4) glycosidic bonds. It contains a large variety of functional groups, and further studies are needed to determine in what form or position these functional groups are connected and how to interact with molecules in the body to regulate body functions. The chemical composition and structure of SFPs vary significantly due to the species of seaweeds, their origin, growth environment, developmental stage, harvesting season, extraction and purification techniques, and measurement methods. Harmonizing these influencing factors to determine the unique composition and structure of polysaccharides is quite challenging.

Regarding the extraction and purification process, the traditional hot-water extraction method is deemed inefficient. While the acid and alkali extraction methods are simple and easy to operate, they may cause damage to the structure of polysaccharides. The application of emerging technologies such as ultrasonic-assisted extraction, enzyme extraction, and microwave-assisted extraction greatly improves the extraction efficiency and preserves the natural active structure of polysaccharides to a certain extent. However, further in-depth study is still needed to optimize the parameters of these methods, especially how to effectively protect the functional groups in the structure during the extraction process, as well as to reduce the risk of polysaccharide degradation during the purification process are still issues that need to be addressed in the future. Meanwhile, the combination of multiple extraction methods may become a trend in future research, such as the combination of ultrasonic-assisted and enzyme extraction, which can further improve the extraction efficiency and biological activity of polysaccharides. In addition, the application of chromatographic separation techniques, such as DEAE-52 cellulose and Sephadex G-200, has significantly improved the purification effect, but its high cost and operational complexity have limited the widespread application. Therefore, simpler and more economical purification strategies should be explored in the future.

In terms of biological efficacy, SFPs demonstrated outstanding antioxidant, antitumor, antiviral, and immunomodulatory activities in vitro and in animal models. In particular, their immune-enhancing effects mediated through the TLR4-MAPK-NF-κB signaling pathway and antitumor activity in the regulation of the VEGF/VEGFR2 signaling pathway show potential prospects for medicinal development. However, most current studies have focused on basic pharmacological evaluations. The mechanism of action of SFPs remains partially unclear, particularly regarding their specific target effects at the cellular and molecular levels. Additionally, the metabolites of SFPs and their absorption, distribution, metabolism, and excretion characteristics within the body are not well understood. In addition, most efficacy studies on SFPs are confined to the animal and cellular levels, SFP remains limited in its clinical applications. The lack of systematic clinical validation further restricts its development as a functional food and drug.

In the future, deeper research on the structure of SFPs should be strengthened, and a more comprehensive structural analysis should be carried out through a variety of modern analytical techniques, so as to clarify the correspondence between key active groups and biological functions.

## Figures and Tables

**Table 1:** The chemical compositions of the crude polysaccharides of SF.

Abbreviation	Mw (kDa)	Sugar (%)	Uronic Acid (%)	Protein (%)	Sulfate Radical (%)	Monosaccharide Composition (%) (Molar Ratio)										Reference
						Glc	GlcA	Man	ManA	GalA	Gal	Xyl	Fuc	Rha	GulA	
PSF	278	74.08	27.3	1.65	6.09	3.09	52.75	4.27	ND	6.62	11.74	3.61	17.93	ND	ND	[24]
PSF	289	ND	ND	ND	ND	7.59	ND	14.56	ND	ND	20.25	9.49	48.1	ND	ND	[25]
JHCP	ND	ND	15.1	9.5	0.31	ND	ND	23.69	ND	ND	25.59	ND	46.76	3.14	ND	[12]
SFPs	299	ND	6.48	ND	10.74	ND	ND	20.18	ND	ND	19.3	8.77	51.75	ND	ND	[26]
HFP	58.28 (89.23%) and 7.46 (10.77%)	73.86	32.62	0.51	5.17	6.47	12.07	16.3	ND	ND	18.71	ND	43.91	2.54	ND	[27]
HFPs	596.67	37.88	ND	ND	ND	0.35	8.72	5.57	49.05	ND	7.92	2.95	22.29	0.29	2.51	[28]
SFP	255.83	83.25	12.80	1.42	7.81	14.93	23.76	3.99	ND	26.25	6.56	1.71	15.3	7.52	ND	[18]
SFP	229	42.69	ND	ND	25.69	ND	ND	ND	ND	ND	ND	ND	19.45	ND	ND	[29]
SFP	51.8	44.52	23.26	1.15	7.91	1.4	2.3	2.5	29	ND	15.2	7.7	25.9	ND	16.1	[30]
SFPs	698.3	80.3	0.17	1	ND	100	ND	ND	ND	ND	ND	ND	ND	ND	ND	[31]
	95.5	63.73	7.71	1.03	11.12	10.5	15	13	ND	ND	11.5	ND	50	ND	ND	
	229.5	69.23	2.34	2.35	35.08	ND	ND	ND	ND	ND	19.4	ND	80.6	ND	ND	
SFP	224	58.10	ND	1.01	13.18	1.5	15.35	9	ND	ND	18.4	5.9	43.2	3.5	ND	[32]
PSF	987	41.9	27.23	2.4	14.72	10.8	ND	3.7	ND	ND	23.6	5.4	56.5	ND	ND	[33]
DPSF	407	54.88	20.15	1.6	16.38	5.4	ND	6.8	ND	ND	29.3	9.5	49	ND	ND	
CDPSF	ND	51.1	24.3	ND	5.94	2	ND	10.1	ND	ND	29.3	6.1	52.5	ND	ND	[21]
HCDPSF	ND	47.2	21.3	ND	5.56	1.3	ND	12.8	ND	ND	26.6	6.4	52.9	ND	ND	
SFPs	299	ND	6.48	ND	10.74	ND	ND	20.18	ND	ND	19.3	8.77	51.75	ND	ND	[34]
SFP	227	41.88	ND	ND	24.73	0.78	12.14	17.31	ND	ND	25.84	7.49	23.51	12.92	ND	[35]
P1	289	20.73	74.04	0.68	1	ND	ND	ND	69.6	ND	ND	ND	9.07	ND	21.32	[36]
ESFP1	ND	68.2	10.1	1.74	8.76	3.9	ND	22.1	ND	ND	20.8	8.5	44.7	ND	ND	[22]
ESFP2	ND	65.9	7.92	1.8	8.89	3.4	ND	23.2	ND	ND	23.1	7.8	42.5	ND	ND	
ESFP3	ND	67	6.72	1.9	10	3.7	ND	19.4	ND	ND	28.2	6.4	42.3	ND	ND	
ESFP4	ND	60.2	1.76	2.14	10.7	3.4	ND	21.4	ND	ND	28.7	5.7	40.8	ND	ND	

Glc: glucose; GlcA: glucuronic acid; Man: mannose; ManA: mannuronic acid; Xyl: xylose; Rha: rhamnose; Gal: galactose; GalA: galacturonic acid; Fuc: fucose; GulA: guluronic acid; PSF: polysaccharides from Sargassum fusiforme; JHCP: Jeju hijiki crude polysaccharides; SFPs: Sargassum fusiforme polysaccharides; HFP: Hizikia fusiforme polysaccharide; HFPs: polysaccharides from Hizikia fusiforme; DPSF: degraded polysaccharides from Sargassum fusiforme; CDPSF: carboxymethylated degraded polysaccharides from Sargassum fusiforme; HCDPSF: hydroxamated derivatives carboxymethylated degraded polysaccharides from Sargassum fusiforme; P1: algal polysaccharide was isolated from Sargassum fusiforme; ESFP: enzymatic hydrolysate of the crude polysaccharide extracted from Sargassum fusiforme; ND: not detected.

**Table 2:** The different extraction methods of SFPs.

Extraction Method	Advantages	Disadvantages	Environmental Impact	Reference
Hot-water extraction method	Low cost; Mild extraction conditions; Preservation of polysaccharide activity; Water-based extraction process; Environmentally friendly method.	Risk of polysaccharide degradation with extended high-temperature treatment; Potential introduction of protein and pigment impurities during extraction; Necessity for additional purification steps due to impurities; Relatively low extraction efficiency.	Environmentally friendly, few solvents used	[53]
Acid extraction method	High extraction efficiency; Selective extraction of acidic polysaccharides; Specifically targets glucuronic acid and fucose; Effectively disrupts cell walls; Acidolysis conditions are controllable; Reaction rate is easily adjustable.	Risk of polysaccharide degradation in acidic environments; Potential for structural changes in acidic conditions; Impact on polysaccharide functional activity; Influence on polysaccharide molecular weight; Need for subsequent neutralization treatment; Complexity of the neutralization process.	Improper disposal of acidic waste liquids may pollute the environment	[54]
Alkali extraction method	Effectively destroys seaweed cell walls; High extraction rate achieved; Suitable for extracting complex polysaccharides; Ideal for seaweed polysaccharide extraction.	Alkaline conditions can cause deacetylation of polysaccharides; Polysaccharide molecular degradation may occur in alkaline environments; These changes can impact the functional activity of polysaccharides; Neutralization treatment is necessary after alkaline treatment; The neutralization process can be complex and challenging to perform.	Alkaline waste liquids may pose a risk to the environment	[55]
Ultrasonic extraction method	High extraction efficiency; Ultrasonic extraction improves cell fragmentation; Maintains the integrity of polysaccharide structure; Reduces energy consumption; Simplifies the operation process.	Equipment cost is high; Ultrasound risks mechanical degradation of polysaccharides; Degradation can affect molecular weight distribution; Biological activity may be compromised; Extraction temperature requires strict regulation; Extraction time necessitates precise control.	Environmentally friendly, low energy consumption, no chemicals	[56]
Microwave extraction method	Fast extraction speed; Enhances extraction efficiency; Uniform microwave heating; Prevents local overheating; Low solvent consumption; Easy control of extraction conditions.	Risk of polysaccharide degradation due to high microwave temperatures; Particular risk for structurally unstable polysaccharides; High cost of microwave equipment; Need for optimization of reaction conditions.	Higher energy consumption, but no solvent pollution to the environment.	[57]
Enzyme extraction method	Mild conditions preserve polysaccharide structure and biological activity; Selective decomposition of cell walls to extract specific polysaccharides; High purity extraction results in reduced impurities.	High selectivity requirements for enzymes; Need for optimization based on specific polysaccharide compositions; Expensive enzyme preparations; Time-consuming digestion process; Complex post-treatment and purification steps for enzymes.	Environmentally friendly, no solvent or waste liquid discharge	[58]
